# Supplementary material for: Affinity gaps among B cells in germinal centers drive the selection of MPER precursors
Source: Nat Immunol. 2024 May 30;25(6):1083–96. doi: 10.1038/s41590-024-01844-7 (PMC11147770; doi:10.1038/s41590-024-01844-7)
Supplement: Supplementary file 1 — Supplementary Tables 1 and 2. [file 41590_2024_1844_MOESM1_ESM.pdf]

nature immunology

Supplementary information
https://doi.org/10.1038/s41590-024-01844-7

Affinity gaps among B cells in germinal centers drive the selection of MPER precursors

In the format provided by the authors and unedited

Table of Contents

Contained within the primary supplemental pdf:

Supplemental Tables

Supplementary Table 1. List of index array and index primers.
Supplementary Table 2. X-ray data collection and refinement statistics.

Uploaded as a separate excel file:

Supplementary Table 3. Reagents and tools, related to Methods.

Table S1: List of Index primer array and index primers used for single cell NGS Sequencing

Index Primer Array

		701	702	703	704	705	706	707	708	709	710	711	712
A	501	701-501	702-501	703-501	704-501	705-501	706-501	707-501	708-501	709-501	710-501	711-501	712-501
B	502	701-502	702-502	703-502	704-502	705-502	706-502	707-502	708-502	709-502	710-502	711-502	712-502
C	503	701-503	702-503	703-503	704-503	705-503	706-503	707-503	708-503	709-503	710-503	711-503	712-503
D	504	701-504	702-504	703-504	704-504	705-504	706-504	707-504	708-504	709-504	710-504	711-504	712-504
E	505	701-505	702-505	703-505	704-505	705-505	706-505	707-505	708-505	709-505	710-505	711-505	712-505
F	506	701-506	702-506	703-506	704-506	705-506	706-506	707-506	708-506	709-506	710-506	711-506	712-506
G	507	701-507	702-507	703-507	704-507	705-507	706-507	707-507	708-507	709-507	710-507	711-507	712-507
H	508	701-508	702-508	703-508	704-508	705-508	706-508	707-508	708-508	709-508	710-508	711-508	712-508

Index Primer List—List of index primers used for single cell NGS sequencing.

701	Illumina ID - D701	CAAGCAGAAGACGGCATACGAGATCGAGTAATGTGACTGGAGTTCAGACGTGTGCTCTTCCGATCT
702	Illumina ID - D702	CAAGCAGAAGACGGCATACGAGATTCTCCGGAGTGACTGGAGTTCAGACGTGTGCTCTTCCGATCT
703	Illumina ID - D703	CAAGCAGAAGACGGCATACGAGATAATGAGCGGTGACTGGAGTTCAGACGTGTGCTCTTCCGATCT
701	Illumina ID - D704	CAAGCAGAAGACGGCATACGAGATGGAATCTCGTGACTGGAGTTCAGACGTGTGCTCTTCCGATCT
702	Illumina ID - D705	CAAGCAGAAGACGGCATACGAGATTTCTGAATGTGACTGGAGTTCAGACGTGTGCTCTTCCGATCT
703	Illumina ID - D706	CAAGCAGAAGACGGCATACGAGATACGAATTCGTGACTGGAGTTCAGACGTGTGCTCTTCCGATCT
701	Illumina ID - D707	CAAGCAGAAGACGGCATACGAGATAGCTTCAGGTGACTGGAGTTCAGACGTGTGCTCTTCCGATCT
702	Illumina ID - D708	CAAGCAGAAGACGGCATACGAGATGCGCATTAGTGACTGGAGTTCAGACGTGTGCTCTTCCGATCT
703	Illumina ID - D709	CAAGCAGAAGACGGCATACGAGATCATAGCCGGTGACTGGAGTTCAGACGTGTGCTCTTCCGATCT
701	Illumina ID - D710	CAAGCAGAAGACGGCATACGAGATTTCGCGGAGTGACTGGAGTTCAGACGTGTGCTCTTCCGATCT
702	Illumina ID - D711	CAAGCAGAAGACGGCATACGAGATGCGCGAGAGTGACTGGAGTTCAGACGTGTGCTCTTCCGATCT
703	Illumina ID - D712	CAAGCAGAAGACGGCATACGAGATCTATCGCTGTGACTGGAGTTCAGACGTGTGCTCTTCCGATCT
501	Illumina ID - D501	AATGATACGGCGACCACCGAGATCTACACTATAGCCTACACTCTTTCCCTACACGACGCTCTTCCGATCT
502	Illumina ID - D502	AATGATACGGCGACCACCGAGATCTACACATAGAGGCACACTCTTTCCCTACACGACGCTCTTCCGATCT
503	Illumina ID - D503	AATGATACGGCGACCACCGAGATCTACACCCTATCCTACACTCTTTCCCTACACGACGCTCTTCCGATCT
504	Illumina ID - D504	AATGATACGGCGACCACCGAGATCTACACGGCTCTGAACACTCTTTCCCTACACGACGCTCTTCCGATCT
505	Illumina ID - D505	AATGATACGGCGACCACCGAGATCTACACAGGCGAAGACACTCTTTCCCTACACGACGCTCTTCCGATCT
506	Illumina ID - D506	AATGATACGGCGACCACCGAGATCTACACTAATCTTAACACTCTTTCCCTACACGACGCTCTTCCGATCT
507	Illumina ID - D507	AATGATACGGCGACCACCGAGATCTACACCAGGACGTACACTCTTTCCCTACACGACGCTCTTCCGATCT
508	Illumina ID - D508	AATGATACGGCGACCACCGAGATCTACACGTACTGACACACTCTTTCCCTACACGACGCTCTTCCGATCT

Table S2. X-ray data collection and refinement statistics

**Data collection**	Ab 45.1 + GT10.2	Ab 45.2 + GT10.2 glycan KO
Beamline	ALS-5.0.1	APS 23-ID-B
Wavelength (Å)	0.97741	1.0337
Space group	P 3_1 2 1	P 1
Unit cell parameters		
a, b, c (Å)	197.0, 197.0, 93.2	37.6, 46.6, 178.4
α, β, γ (°)	90, 90, 120	86.7, 86.8, 77.0
Resolution (Å) [a]	50-3.00 (3.05-3.00)	50-2.07 (2.11-2.07)
Unique reflections [a]	41,850 (3,894)	63,266 (6,109)
Redundancy [a]	7.5 (7.5)	3.0 (1.9)
Completeness (%) [a]	100 (100)	88.6 (88.0)
$\langle I/\sigma_I \rangle$ [a]	5.9 (0.3)	8.9 (2.0)
R_{sym}[b] (%) [a]	30.5 (>100)	14.5 (48.6)
R_{pim}[b] (%) [a]	12.7 (>100)	9.7 (40.5)
$CC_{1/2}$[c] (%) [a]	97.3 (30.8)	98.3 (83.6)
**Refinement statistics**		
Resolution (Å)	49.2-3.00	44.5-2.07
Reflections (work)	40,528	62,857
Reflections (test)	2,058	3,069
R_{cryst}[d] / R_{free}[e] (%)	26.8/29.3	25.0/29.1
No. of copies in ASU	1	2
No. of atoms	4,639	9,996
Fab	3,382	6,818
Scaffold	1,257	2,486
Solvent	N/A	692
Average *B*-values ($Å^2$)	76	31
Fab	71	29
Scaffold	90	34
Solvent	N/A	38
Wilson *B*-value ($Å^2$)	55	24
**RMSD from ideal geometry**		
Bond length (Å)	0.002	0.003
Bond angle (°)	0.60	0.65
**Ramachandran statistics (%)** [f]		
Favored	95.1	98.0
Outliers	0.00	0.08
**PDB code**	9BDH	9BDI

[a] Numbers in parentheses refer to the highest resolution shell.

[b] $R_{sym} = \Sigma_{hkl} \Sigma_i | I_{hkl,i} - \langle I_{hkl} \rangle | / \Sigma_{hkl} \Sigma_i I_{hkl,i}$ and $R_{pim} = \Sigma_{hkl} (1/(n-1))^{1/2} \Sigma_i | I_{hkl,i} - \langle I_{hkl} \rangle | / \Sigma_{hkl} \Sigma_i I_{hkl,i}$, where $I_{hkl,i}$ is the scaled intensity of the ith measurement of reflection h, k, l, $\langle I_{hkl} \rangle$ is the average intensity for that reflection, and *n* is the redundancy.

[c] $CC_{1/2}$ = Pearson correlation coefficient between two random half datasets.

[d] $R_{cryst} = \Sigma_{hkl} | F_o - F_c | / \Sigma_{hkl} | F_o | \times 100$, where F_o and F_c are the observed and calculated structure factors, respectively.

[e] R_{free} was calculated as for R_{cryst}, but on a test set comprising 5% of the data excluded from refinement.

[f] From MolProbity (*71*).
