# Supplementary material for: Affinity gaps among B cells in germinal centers drive the selection of MPER precursors
Source: Nat Immunol. 2024 May 30;25(6):1083–96. doi: 10.1038/s41590-024-01844-7 (PMC11147770; doi:10.1038/s41590-024-01844-7)
Supplement: Supplementary file 2 — Reporting Summary [file 41590_2024_1844_MOESM2_ESM.pdf]

nature portfolio

Corresponding author(s): William R. Schief and Facundo D. Batista

Last updated by author(s): Apr 12, 2024

Reporting Summary

Nature Portfolio wishes to improve the reproducibility of the work that we publish. This form provides structure for consistency and transparency in reporting. For further information on Nature Portfolio policies, see our Editorial Policies and the Editorial Policy Checklist.

Statistics

For all statistical analyses, confirm that the following items are present in the figure legend, table legend, main text, or Methods section.

n/a	Confirmed	
☐	☒	The exact sample size (*n*) for each experimental group/condition, given as a discrete number and unit of measurement
☐	☒	A statement on whether measurements were taken from distinct samples or whether the same sample was measured repeatedly
☐	☒	The statistical test(s) used AND whether they are one- or two-sided<br>*Only common tests should be described solely by name; describe more complex techniques in the Methods section.*
☐	☒	A description of all covariates tested
☐	☒	A description of any assumptions or corrections, such as tests of normality and adjustment for multiple comparisons
☐	☒	A full description of the statistical parameters including central tendency (e.g. means) or other basic estimates (e.g. regression coefficient) AND variation (e.g. standard deviation) or associated estimates of uncertainty (e.g. confidence intervals)
☐	☒	For null hypothesis testing, the test statistic (e.g. *F*, *t*, *r*) with confidence intervals, effect sizes, degrees of freedom and *P* value noted<br>*Give P values as exact values whenever suitable.*
☒	☐	For Bayesian analysis, information on the choice of priors and Markov chain Monte Carlo settings
☒	☐	For hierarchical and complex designs, identification of the appropriate level for tests and full reporting of outcomes
☒	☐	Estimates of effect sizes (e.g. Cohen's *d*, Pearson's *r*), indicating how they were calculated

Our web collection on statistics for biologists contains articles on many of the points above.

Software and code

Policy information about availability of computer code

Data collection	BD FACSDiva™ Software, Biotek Gen5
Data analysis	FlowJo, Prism 9.5.1, Geneious Prime, FLASh, MiXCR, Microsoft Office

For manuscripts utilizing custom algorithms or software that are central to the research but not yet described in published literature, software must be made available to editors and reviewers. We strongly encourage code deposition in a community repository (e.g. GitHub). See the Nature Portfolio guidelines for submitting code & software for further information.

Data

Policy information about availability of data

All manuscripts must include a data availability statement. This statement should provide the following information, where applicable:

- Accession codes, unique identifiers, or web links for publicly available datasets
- A description of any restrictions on data availability
- For clinical datasets or third party data, please ensure that the statement adheres to our policy

BCR sequences at PP617372–PP617659 and PP617660–PP618659 and crystal structures at 9BDH & 9BDI. No original code is reported in this manuscript. Any additional information required to reanalyze the data reported in this paper is available from the lead contact (F.D.B.) upon request.

Research involving human participants, their data, or biological material

Policy information about studies with human participants or human data. See also policy information about sex, gender (identity/presentation), and sexual orientation and race, ethnicity and racism.

Reporting on sex and gender	No human participants, data, or biological material are included.
Reporting on race, ethnicity, or other socially relevant groupings	No human participants, data, or biological material are included.
Population characteristics	No human participants, data, or biological material are included.
Recruitment	No human participants, data, or biological material are included.
Ethics oversight	No human participants, data, or biological material are included.

Note that full information on the approval of the study protocol must also be provided in the manuscript.

Field-specific reporting

Please select the one below that is the best fit for your research. If you are not sure, read the appropriate sections before making your selection.

☒ Life sciences ☐ Behavioural & social sciences ☐ Ecological, evolutionary & environmental sciences

For a reference copy of the document with all sections, see nature.com/documents/nr-reporting-summary-flat.pdf

Life sciences study design

All studies must disclose on these points even when the disclosure is negative.

Sample size	All experimental groups and numbers are specified in the figures legends; sample sizes were determined based on approaches used in the literature for similar immunogens/models, as described in the Methods.
Data exclusions	No data was excluded.
Replication	All major assays were repeated at least once (as noted in legends) to verify their reproducibility and were successful.
Randomization	Mice for experimental and control groups derive from the same colonies and were randomly assigned to groups.
Blinding	Blinding was not employed, as the first author's involvment from mouse development, maintenance, injection, and analysis rendered analytical blinding infeasible.

Reporting for specific materials, systems and methods

We require information from authors about some types of materials, experimental systems and methods used in many studies. Here, indicate whether each material, system or method listed is relevant to your study. If you are not sure if a list item applies to your research, read the appropriate section before selecting a response.

Materials & experimental systems

n/a	Involved in the study	
☐	☒	Antibodies
☒	☐	Eukaryotic cell lines
☒	☐	Palaeontology and archaeology
☐	☒	Animals and other organisms
☒	☐	Clinical data
☒	☐	Dual use research of concern
☒	☐	Plants

Methods

n/a	Involved in the study	
☒	☐	ChIP-seq
☐	☒	Flow cytometry
☒	☐	MRI-based neuroimaging

Antibodies

Antibodies used	All antibodies used in this study have been listed with their catalog numbers and supplier in the Reagents and Tools Table S3 in the manuscript; dilutions are described in the Methods section.

Validation	All antibodies deployed here have been previously used effectively in similarly-generated KI mice (e.g., Melzi et al., Immunity 2022; Wang et al., EMBO J 2021; Kratochvil et al., Immunity 2021; Tas et al., Immunity 2022).

Animals and other research organisms

Policy information about studies involving animals; ARRIVE guidelines recommended for reporting animal research, and Sex and Gender in Research

Laboratory animals	B6.SJL-Ptprc a Pepc b /BoyJ mice (CD45.1+/+) 8–12 weeks of age and KI mice described in text housed in a facility at ambient 68F/40% humidity on a 12:12 light cycle.
Wild animals	No wild animals were used.
Reporting on sex	Both male and female donor mice were used for adoptive transfers. All transfers and immunizations were done in male CD45.1+/+ mice due to risk of rejection of cells from male donor to female recipient mice.
Field-collected samples	No field collections were performed.
Ethics oversight	All experiments were performed under the approval by the AALAC-accredited Institutional Animal Care and Use Committee (IACUC) of Harvard University and the Massachusetts General Hospital (MGH) (Animal Study Protocols 2016N000022 and 2016N000286).

Note that full information on the approval of the study protocol must also be provided in the manuscript.

Flow Cytometry

Plots

Confirm that:

☒ The axis labels state the marker and fluorochrome used (e.g. CD4-FITC).

☒ The axis scales are clearly visible. Include numbers along axes only for bottom left plot of group (a 'group' is an analysis of identical markers).

☒ All plots are contour plots with outliers or pseudocolor plots.

☒ A numerical value for number of cells or percentage (with statistics) is provided.

Methodology

Sample preparation	At selected time points following immunization, whole spleens were mechanically dissociated to generate single-cell suspensions. ACK lysis buffer was used to remove red blood cells and splenocytes were then resuspended in FACS buffer (2% FBS/PBS), Fc-blocked (clone 2.4G2, BD Biosciences) and stained for viability with Live/Dead Blue (Thermo Fisher Scientific) for 20 min at 4[illegible]C. For surface staining GT9 or GT10 probes (described above), as well as antibodies against CD4-APCeF780, CD8-APC-eF780, Gr-1-APC-eF780, F4/80-APC-eF780, B220-B510, CD95-PE-Cy7, CD38-A700, CD45.1-PerCPCy5.5, CD45.2-PE, IgD-BV786, IgM-BUV395 and IgG1-BV421, were used.
Instrument	Cells were acquired by a BD LSRFortessa (BD Biosciences) for flow cytometric analysis and sorted using a BD FACS Aria II instrument (BD Biosciences).
Software	BD FACSDiva™ Software used for data collection and analysis. FlowJo used for data analysis.
Cell population abundance	Live dead staining was used to confirm sample quality. Population gates described in the text were then used to characterize the abundance of each cell fraction.
Gating strategy	The gating strategies are S3D, wherein the gating strategy shows identification of GC B cells (CD95hiCD38lo) (pregated on B220+Live/Dump-) and the proportion of CD45.1+ and CD45.2+ B cells in GCs followed by the percentage of epitope specific cells (GT9++KO-), the same gating has been used for all germinal center analysis, and S10E which shows gating strategy identification of memory B cells as CD38+CD95-IgD- B220+CD45.2+GT10++ B cells.

☒ Tick this box to confirm that a figure exemplifying the gating strategy is provided in the Supplementary Information.
